# Alpha-actinin-binding antibodies in relation to systemic lupus erythematosus and lupus nephritis

**DOI:** 10.1186/ar2070

**Published:** 2006-10-24

**Authors:** Andrea Becker-Merok, Manar Kalaaji, Kaia Haugbro, Cathrin Nikolaisen, Kirsten Nilsen, Ole Petter Rekvig, Johannes C Nossent

**Affiliations:** 1 Department of Rheumatology, Institute of Clinical Medicine, University of Tromsø, Breivika, N-9037 Tromsø, Norway; 2 Department of Biochemistry, Institute of Medical Biology, University of Tromsø, Breivika, N-9037 Tromsø, Norway; 3 Department of Rheumatology, University Hospital of North Norway, Tromsø, Breivika, N-9038 Tromsø, Norway

## Abstract

This study investigated the overall clinical impact of anti-α-actinin antibodies in patients with pre-selected autoimmune diseases and in a random group of anti-nuclear antibody (ANA)-positive individuals. The relation of anti-α-actinin antibodies with lupus nephritis and anti-double-stranded DNA (anti-dsDNA) antibodies represented a particular focus for the study. Using a cross-sectional design, the presence of antibodies to α-actinin was studied in selected groups, classified according to the relevant American College of Rheumatology classification criteria for systemic lupus erythematosus (SLE) (*n *= 99), rheumatoid arthritis (RA) (*n *= 68), Wegener's granulomatosis (WG) (*n *= 85), and fibromyalgia (FM) (*n *= 29), and in a random group of ANA-positive individuals (*n *= 142). Renal disease was defined as (increased) proteinuria with haematuria or presence of cellular casts. Sera from SLE, RA, and Sjøgren's syndrome (SS) patients had significantly higher levels of anti-α-actinin antibodies than the other patient groups. Using the geometric mean (± 2 standard deviations) in FM patients as the upper cutoff, 20% of SLE patients, 12% of RA patients, 4% of SS patients, and none of the WG patients were positive for anti-α-actinin antibodies. Within the SLE cohort, anti-α-actinin antibody levels were higher in patients with renal flares (*p *= 0.02) and correlated independently with anti-dsDNA antibody levels by enzyme-linked immunosorbent assay (*p *< 0.007) but not with other disease features. In the random ANA group, 14 individuals had anti-α-actinin antibodies. Of these, 36% had SLE, while 64% suffered from other, mostly autoimmune, disorders. Antibodies binding to α-actinin were detected in 20% of SLE patients but were not specific for SLE. They correlate with anti-dsDNA antibody levels, implying *in vitro *cross-reactivity of anti-dsDNA antibodies, which may explain the observed association with renal disease in SLE.

## Introduction

A wide spectrum of organ non-specific autoantibodies can be detected in sera of patients with systemic lupus erythematosus (SLE) [1]. Although the clinical significance of many of these autoantibodies remains unclear, anti-double-stranded DNA (anti-dsDNA) antibodies (Abs) are among the most SLE-specific autoantibodies and are also involved in the pathogenesis of lupus nephritis (LN) [2-7]. Given the consequences of LN in terms of morbidity, mortality, and treatment-related toxicity, increased knowledge on the pathophysiology of LN is needed to develop therapeutic interventions that are more rational. Intraglomerular immune complex depositions are a hallmark of LN, and anti-dsDNA Abs can be eluted from affected kidneys in both human and experimental LN [8-10]. The glomerular target structures for anti-dsDNA Abs, however, are still controversial, and to determine structures that *de facto *bind Abs *in vivo *is more important than to determine potential cross-reactions of nephritogenic autoantibodies.

Several models explain anti-dsDNA Ab binding in the glomeruli. In one model, Ab binds to externalised nucleosomes present in basement membranes and the mesangium of glomeruli [11,12], whereas other models focus on Ab binding to basement membrane constituents, either by specific recognition or by cross-reaction of anti-dsDNA Abs [5,13]. Recent reports have indicated that anti-dsDNA Abs may specifically cross-react with intraglomerular, extracellular α-actinin in patients with LN [14-19]. The rod-shaped α-actinin proteins are central to the organisation of the cytoskeleton as they bind and crosslink actin [20]. In the kidney, α-actinin has been detected in mesangial cells, podocytes, capillaries, and larger blood vessels [20-23], where it plays a role in the formation of adhesion receptors [24-27] that link the cytoskeleton with the extracellular matrix [28-31]. There is also evidence that membrane-associated α-actinin is accessible on the surface of mesangial cells [15,18], and the increased glomerular α-actinin expression after epithelial podocyte confluence and the occurrence of proteinuria suggest a role for α-actinin in renal pathophysiology [32,33]. In view of the above, it seems improbable that the intraglomerular presence of (non-muscle) α-actinin would be a specific occurrence in patients with LN, although an Ab response to α-actinin may still be specific for SLE and contribute to LN.

Therefore, the presence of α-actinin-binding Abs was investigated in patients with various autoimmune systemic inflammatory diseases, including SLE. Furthermore, because anti-dsDNA Abs are thought to mediate their nephritogenic potential in patients with SLE through cross-reactive α-actinin binding, we analysed the associations between α-actinin binding and clinical and immunological manifestations in patients with SLE in more detail.

## Materials and methods

### Patients and definitions

Abs to α-actinin were analysed in a cross-sectional study in two different sets of patients. First, patients were selected based on scientific classification according to the relevant American College of Rheumatology classification criteria for SLE (*n *= 99), rheumatoid arthritis (RA) (*n *= 68), Wegener's granulomatosis (WG) (*n *= 85), and fibromyalgia (FM) (*n *= 29) [34-37]. Patients included in disease registries, which are approved by the Regional Ethics Committee, gave informed written consent. In addition, Abs to α-actinin were analysed in 142 consecutively collected anti-nuclear Ab (ANA)-positive sera, in which subsequent clinical diagnoses were settled without the knowledge of the serological analyses [38]. Detailed information on these cohorts has been published before [38-41], and demographic data for the different subgroups are given in Table 1. Disease activity in patients with SLE was determined by the calculation of a Systemic Lupus Erythematosus Disease activity index (SLEDAI), based upon clinical findings in the 2 weeks prior to sample collection. Renal flares were defined according to the SLEDAI definition of new onset or recent increase (>0.5 g per 24 hours) of proteinuria. In eight patients, renal biopsy verified the LN diagnosis, whereas three patients were treated for a relapse of earlier (1 to 3 years) biopsy-confirmed LN. In these three patients, LN diagnosis was not confirmed by biopsy and was based solely on the SLEDAI definition.

### ANA screening assay

ANAs were determined by a screening enzyme-linked immunosorbent assay (ELISA) (Phadia GmbH, Freiburg, Germany, formerly Pharmacia Diagnostics), using the assay protocol recommended by the manufacturer. Cutoff was controlled as described previously [42] and verified the cutoff suggested by the manufacturer.

### Anti-DNA Ab assays

Abs to dsDNA by ELISA were determined and quantified by a widely used commercially available and internationally validated anti-DNA Ab kit (Varelisa; Phadia GmbH). The cutoff values were determined locally through a continuously running internal quality assessment program, as recently described [42]. Lot-to-lot variation of analytical ELISA-based kits, relevant to the determination of cutoff values, was examined and adjusted when necessary by internal and external reference Abs. The selected cutoff value agreed with other laboratories participating in national and international quality assessment programs. A result was regarded as positive at greater than or equal to 55 Units for the anti-dsDNA ELISA. This cutoff value is regarded as sufficient to avoid Abs we regard as insignificant and epiphenomenological from insight into their origin and clinical impact and are in line with cutoff values adapted by other laboratories [43].

The fluorescence enzyme immunoassay for anti-dsDNA by EliA test (Phadia GmbH) was processed using UniCap100 (Phadia GmbH) as recommended by the manufacturer [44]. Bound human anti-dsDNA Abs were detected by mouse anti-human Fcγ Ab conjugated with β-galactosidase and 4-methylumbelliferyl-β-D-galactoside as substrate. Washing of the wells was performed using a stringent washing buffer, which dissociates and thereby avoids detection of low-avidity Abs. A six-point standard curve calibrated against World Health Organization reference sera was used for quantitative measurements, and results are given as arbitrary IU, with a positive result defined as greater than or equal to 20 IU.

### Anti-α-actinin assay

Abs to α-actinin were determined by an in-house ELISA test using chicken α-actinin (Sigma-Aldrich, St. Louis, MO, USA) as target antigen, as described recently [45]. All sera were titrated by twofold dilution. Because the values in general were very low, data are presented as optical density (OD)_490 nm _at 1:100 dilution. Goat polyclonal immunoglobulin G anti-α-actinin (Santa Cruz Biotechnology, Inc., Santa Cruz, CA, USA) was used as an intra-assay positive control Ab. A result was defined as positive if the mean OD was higher than the geometric mean (± 2 standard deviations) level of binding of FM sera to α-actinin resulting in a cutoff level of OD 0.133 at 490 nm, if not otherwise stated in the text.

### Statistics

Differences between values for the various groups were analysed with Fisher exact test for dichotomous variables and Kruskal-Wallis test for continuous variables, and correlations were estimated by Spearman rank test coefficient. Nonparametric tests were chosen because of the skewness of the data. To determine the independence of factors with a significant Spearman rank correlation to α-actinin binding, multivariate regression analyses were performed in a step-up method (*p *< 0.1 to enter). All analyses were performed using SPSS version 11.0 (SPSS Inc., Chicago, IL, USA). Resulting two-sided *p *values < 0.05 were considered to indicate significance.

## Results

### Diagnostic impact of anti α-actinin Abs in pre-selected groups or in ANA-positive individuals

The frequency of anti-α-actinin Abs in the pre-selected groups was 5.9% when combining RA and WG patients in one group, whereas the frequency was 20% in the SLE group (Table 1). Although the fourfold-higher frequency in SLE was statistically significant, analysing pre-selected groups of patients might have introduced bias by excluding a wider array of conditions in which Abs to α-actinin potentially may be produced. To test for this, another approach was undertaken with patients selected purely on the basis of a positive ANA test. Because ANA may be present in a wide variety of conditions and also among normal individuals, the bias toward SLE for this approach is insignificant [46].

In the ANA-positive group, 46 (32%) individuals were positive for Abs to α-actinin at the cutoff value of OD_490 nm _at 0.133. These patients demonstrated a large spectrum of disorders, and anti-dsDNA Abs were most frequently but not exclusively found in patients with SLE (Table 2). Notwithstanding this wide disease spectrum, there was a significant correlation between anti-dsDNA Ab and anti-α-actinin Ab levels present in this ANA-positive cohort (Spearman's rho [Rs] 0.27, *p *= 0.04). Using a more restricted cutoff value (OD_490 nm _of 0.2 after subtraction of background binding in albumin-coated wells), 14 patients remained positive and these patients were later diagnosed with the following: SLE (*n *= 5), Sjøgren's syndrome (SS) (*n *= 3), discoid lupus erythematosus (*n *= 1), RA (*n *= 1), arthralgia (*n *= 1), urinary tract infection (*n *= 1), autoimmune hepatitis (*n *= 1), and unclassified connective tissue disease (*n *= 1). This confirms that anti-α-actinin Abs occur among random, ANA-positive, non-SLE patients [45].

There were significant differences in the levels of anti-α-actinin Abs in the selected disease groups (Table 1). Patients with SLE and RA had higher OD values than patients with WG and also compared with the randomised ANA-positive patients (*p *= 0.01). The differences between the SLE and RA groups were not significant as was the case also between WG and ANA-positive patients (all *p *values > 0.3). In a sub-analysis of ANA-positive patients, anti-α-actinin Ab levels were also higher in those with SLE, RA, and SS compared with patients with other disorders (*p *= 0.05, data not shown), although differences between SLE, RA, and SS patients were not significant.

### Correlation between α-actinin binding and disease features in SLE cohort

**Figure 1:**
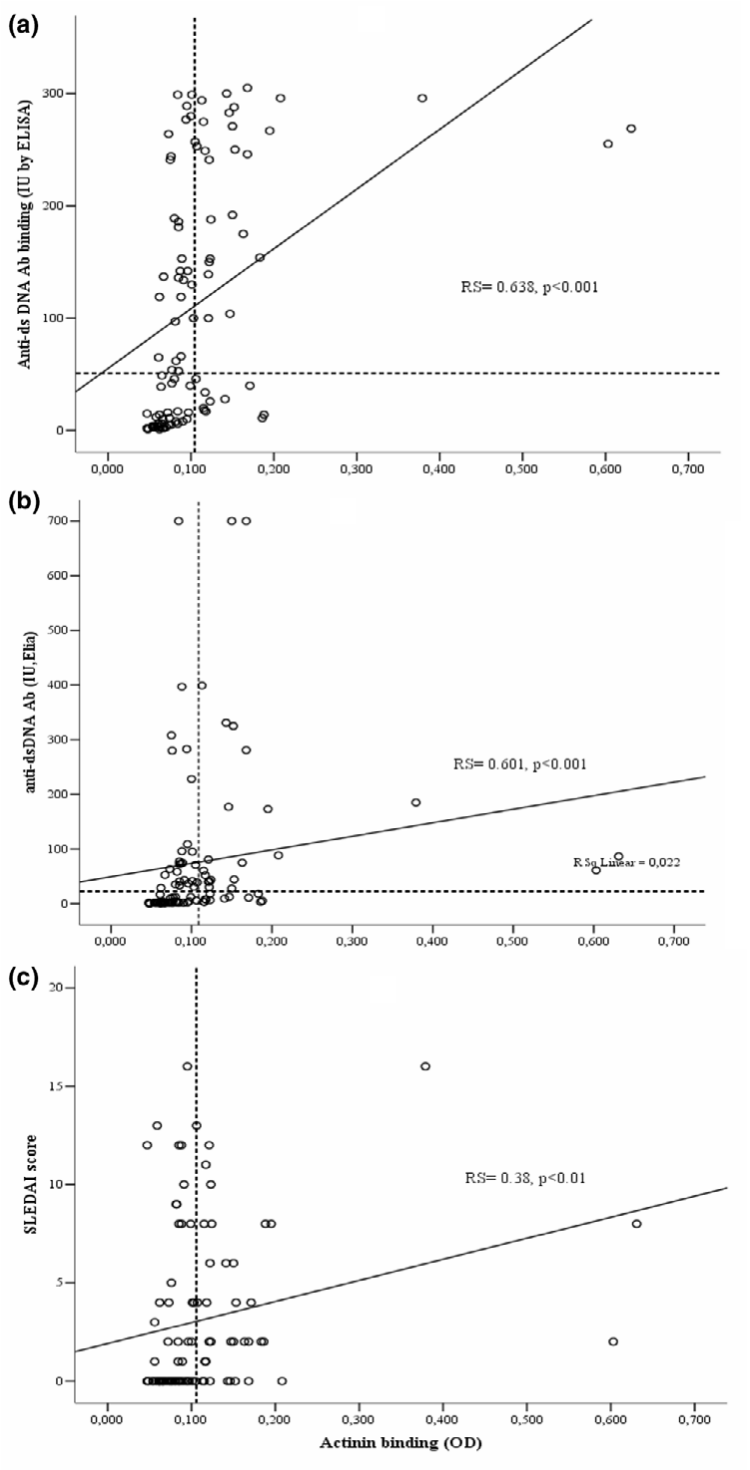
Scatterplots representing the relationship between α-actinin antibody (Ab) binding and levels of anti-double-stranded DNA (anti-dsDNA) Abs in the pre-selected diagnostic groups. The relationship of α-actinin Ab binding with anti-dsDNA Abs detected by enzyme-linked immunosorbent assay (ELISA) **(a) **or by EliA assay **(b) **and with overall disease activity (SLEDAI) **(c)**. Broken lines indicate cutoff levels for the respective assays (see Materials and methods for analytical details). EliA, fluorescence enzyme immunoassay test for anti-dsDNA (Phadia GmbH); OD, optical density; Rs, Spearman's rho; SLEDAI, Systemic Lupus Erythematosus Disease activity index.

Levels of anti-dsDNA Abs in both the ELISA and the EliA assay as well as clinical disease activity (SLEDAI) scores were significantly correlated with the presence of anti-α-actinin Abs (Table 3, Figure 1). This remained unaltered after Bonferroni correction and also when excluding the three outliers (Rs 0.35, *p *= 0.001). In addition, C-reactive protein (CRP) levels, erythrocyte sedimentation rate, damage index (*p *< 0.05), and age (*p *= 0.051) correlated with anti-α-actinin Abs prior to Bonferroni correction, whereas no correlation was seen with quantitative renal features such as proteinuria or serum creatinin levels (Table 3). In a multivariate regression analysis, only anti-dsDNA Abs detected by ELISA remained independently correlated with α-actinin binding (Table 4).

### Correlation between anti-α-actinin Abs and renal flares in SLE cohort

**Figure 2:**
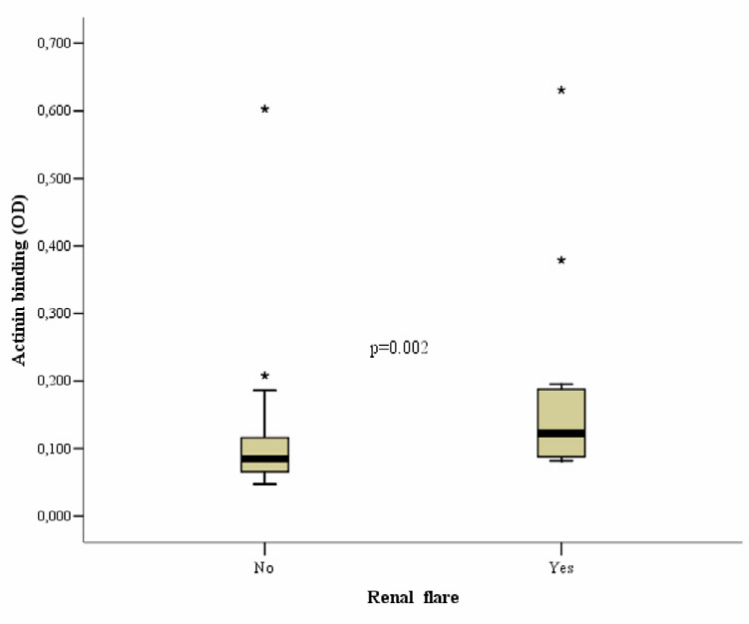
Box plot of the optical density (OD) of α-actinin binding in patients from pre-selected systemic lupus erythematosus group with and without renal flares. Thick bars indicate median values, and boxes border the interquartile range. Asterisks represent outliers.

Renal disease flares, as defined in Materials and methods, were present in 14 patients in the pre-selected SLE group. These patients had higher levels of anti-α-actinin Ab binding (median OD_490 nm _180 versus 100, *p *= 0.002) (Figure 2) as well as higher SLEDAI scores (9 versus 2, *p *= 0.001) and anti-dsDNA Ab levels by EliA (67.9 versus 12.1 (median), *p *= 0.013) than patients without renal flare, and both CRP and dsDNA Ab levels by ELISA did not differ (*p *values > 0.2; data not shown). Using the standard cutoff value (OD_490 nm _0.133), 43% of patients with LN were positive for α-actinin Abs versus 17% of SLE patients without nephritis (*p *= 0.034; odds ratio 3.8, confidence interval 1.1 to 12.7), and 71% of patients with LN tested positive for anti-dsDNA Abs (EliA) versus 45% of SLE patients without nephritis (*p *= 0.08). The proportion of patients with positive ELISA anti-dsDNA Ab findings did not differ between both groups (71% versus 49%, *p *= 0.156).

## Discussion

In the present study, a critical analysis of the clinical impact of Abs to α-actinin was performed, with a focus on their diagnostic significance and alleged correlation with LN. To obtain sound information, two principally different analytical models were tested; in one model, pre-selected groups of patients with established diagnosis were analysed, whereas the other implemented a randomised group of patients in which a positive ANA test was the only selection criterion. The wider scope of this two-sided approach increases the reliability of data on the value of diagnostic testing in general and puts the clinical significance of Abs to α-actinin in a broader perspective than prior studies.

In this study, Ab binding of α-actinin was four to five times more prevalent in SLE than in the other pre-selected diagnostic groups. This could indicate that Abs to α-actinin might serve as a diagnostically valuable parameter for SLE. However, in a randomised ANA-positive group, the anti-α-actinin Ab was more prevalent in non-SLE patients than in SLE patients. The 20% prevalence of these Abs in SLE patients together with its low specificity compared with other rheumatologic and noninflammatory diseases indicate that testing for this Ab is not likely to be useful in diagnosing systemic autoimmune disease states. The data demonstrate that the diagnostic power of a given parameter – here, the anti-α-actinin Ab – should ideally be determined in randomised studies and not (only) in selected groups of patients. This is in agreement with results from studies of the diagnostic impact of different analytical methods for anti-dsDNA Abs in the same ANA-positive group of individuals [38].

Within the SLE cohort, α-actinin Abs correlated with features of disease activity, including anti-dsDNA Ab levels. Specifically, renal involvement was associated with higher α-actinin Ab binding, and α-actinin-positive lupus patients were 3.8 times more likely to have renal involvement. Thus, in patients with established SLE, α-actinin Abs may be associated in some way with renal disease.

A specific role for α-actinin-binding Abs in the pathophysiology of (renal) disease in SLE has not yet been defined. The fact that anti-dsDNA Ab presence was the sole independent factor for α-actinin Ab presence in this multivariate analysis, however, provides indirect support for earlier observations of cross-reactions between anti-dsDNA Abs and α-actinin. The demonstration of Abs to α-actinin in eluates from nephritic murine kidneys indicates that this Ab population is present in nephritic glomeruli. However, just as serological profiles of anti-dsDNA Abs do not necessarily predict the development of LN, this also holds true for α-actinin-binding Abs because neither DNA nor α-actinin is normally an available target antigen in the kidney. By analogy with glomerular binding of anti-dsDNA Abs requiring nucleosomes to become accessible in the extra-cellular space, intra-glomerular anti-α-actinin Ab deposition would require the release of α-actinin in extra-cellular space. Recent results from our laboratory demonstrate the presence of extra-cellular, intraglomerular α-actinin in nephritic, but not in healthy, murine glomeruli [45]. Also, even though subgroups of anti-dsDNA Abs may cross-react with α-actinin *in vitro*, this does not support the conclusion that this protein also represents the intra-glomerular target for anti-dsDNA Abs in LN. Thus, the present data do not prove a causal relationship between Abs to α-actinin and nephritis.

Indirect *in vitro *evidence from experimental LN indicates a possible role for cross-reactive binding of anti-dsDNA Abs to intraglomerular antigens in the absence of DNA [15,18], but definite *in vivo *proof is lacking. In contrast, the distribution of glomerular α-actinin did not correlate with the distribution of *in vivo*-bound, glomerular basement membrane-associated autoantibodies in a recent study [45]. These findings suggest that anti-α-actinin Abs mainly constitute an epiphenomena with limited clinical relevance. Confirming a role in monitoring patients with established SLE for renal disease would require proof from longitudinal studies.

Some limitations apply to the findings presented here. Overall, the mean level of Ab binding to α-actinin (by OD) in the disease subgroups was quite low. Both mean levels and cutoff levels reported here are, however, in agreement with other findings on anti α-actinin Ab binding in humans [45,47,48]. Nonetheless, it may be argued that such low OD values are not meaningful, especially as the potential pathophysiological significance of the presence of α-actinin Abs remains unclear. Patients in the ANA-positive cohort were classified according to established guidelines; however, our approach to exclude anti-dsDNA Ab as a criterion may have introduced a bias toward non-SLE cohorts. Also, the prevalence and disease severity of LN and autoantibodies are markedly higher in non-white populations. The exclusive Caucasian make-up of these cohorts makes it difficult to extrapolate our findings to cohorts of different ethnic background. Although our cutoff levels for normal values were based on FM patients, who do not have an inflammatory autoimmune disorder and in whom levels were comparable with those in healthy controls, this nonetheless may have introduced bias in our results.

## Conclusion

The impact of Abs to α-actinin as diagnostic markers for SLE is limited. The association between renal involvement in SLE and the presence of Abs to α-actinin is likely the result of cross-reactive anti-dsDNA Abs. A pathophysiological role for cross-reactivity of anti-dsDNA Abs with extracellular α-actinin *in vivo*, however, is not supported by experimental models for LN.

## Abbreviations

Ab = antibody; ANA = anti-nuclear antibody; anti-dsDNA = anti-double-stranded DNA; CRP = C-reactive protein; EliA = fluorescence enzyme immunoassay test for anti-double-stranded DNA (Phadia GmbH); ELISA = enzyme-linked immunosorbent assay; FM = fibromyalgia; LN = lupus nephritis; OD = optical density; RA = rheumatoid arthritis; Rs = Spearman's rho; SLE = systemic lupus erythematosus; SLEDAI = Systemic Lupus Erythematosus Disease activity index; SS = Sjøgren's syndrome; WG = Wegener's granulomatosis.

## Competing interests

The authors declare that they have no competing interests.

## Authors' contributions

OPR and HN worked out the study design. ABM, CN, and HN conducted the clinical data collection. MK, KH, CN, KN, OPR, and HN performed the laboratory analyses. ABM, MK, HN, and OPR participated in the data analysis and statistics. ABM, OPR, and HN contributed to the writing of the manuscript. All authors read and approved the final version.

## Figures and Tables

**Table 1:** Demographics and α-actinin binding in the various disease groups

Disease group	Female/male ratio (percentage)	Age in years (range)	OD α-actinin binding (range)	No. (percentage) with positive α-actinin binding
RA (*n *= 68)	65/35	67.7 (24 to 85)	0.102 (0.06 to 0.26)^a^	8 (12)^a^
SLE (*n *= 99)	92/8	35.7 (15 to 73)	0.112 (0.05 to 0.63)^a^	20 (20)^a^
WG (*n *= 85)	57/43	49.1 (17 to 76)	0.053 (0.04 to 0.08)	0 (0)
ANA-positive (*n *= 142)	63/37	52.2 (6 to 78)	0.09 (0.06 to 0.90)	46 (32)^a^

^a^Indicates significant difference compared with WG category. Numbers represent mean values (range) unless otherwise indicated. ANA, anti-nuclear antibody; OD, optical density; RA, rheumatoid arthritis; SLE, systemic lupus erythematosus; WG, Wegener's granulomatosis.

**Table 2:** Results of anti-dsDNA testing according to disease classification in ANA-positive subjects

	Anti-dsDNA (ELISA)		Anti-dsDNA (EliA)	
	
Disease classification	Percentage positive	Titre (IU) (SD)	Percentage positive	Titre (IU) (SD)
SLE (*n *= 39)	79	171 (51)	44	138 (196)
SS (*n *= 22)	18	152 (70)	12	89 (56)
RA (*n *= 17)	29	200 (0)	6	48 (2)
UCTD (*n *= 9)	22	194 (8)	22	59 (0.2)
Various (*n *= 57)^a^	35	143 (82)	22	36 (49)

Values represent mean values (SD) unless otherwise indicated. ^a^Includes patients with viral hepatitis, angina pectoris, apnoea syndrome, arthralgia/lumbago, asthma/bronchitis, cataract/cornea bleeding, claudicatio intermittens, cerebrovascular accident, epilepsy, erythematosus nodosum, fibromyalgia, haematuria, headache, hypothyroidism, urinary tract infections, solid cancer, menisc rupture, migraine, myasthenia gravis, panniculitis, psoriasis, rash, urine incontinence, observation, periorbital edema, and Stevens-Johnson syndrome. Anti-dsDNA, anti-double-stranded DNA; EliA, fluorescence enzyme immunoassay test for anti-double-stranded DNA (Phadia GmbH); ELISA, enzyme-linked immunosorbent assay; RA, rheumatoid arthritis; SD, standard deviation; SLE, systemic lupus erythematosus; SS, Sjøgren's syndrome; UCTD, unclassified connective tissue disease.

**Table 3:** Correlation between α-actinin-binding antibody levels and clinical findings in patients with SLE

Disease feature	Spearman's rho	*p *value
Anti-dsDNA Ab ELISA (IU/ml)	0.638	0.0001^a^
Anti-dsDNA Ab EliA (IU/ml)	0.601	0.0001^a^
Total SLEDAI score	0.378	0.0001^a^
ESR	0.254	0.035
CRP (mg/l)	0.236	0.048
C3 (mg/l)	-0.102	0.522
C4 (mg/l)	-0.078	0.623
Age (years)	-0.197	0.051
Creatinin clearance (ml/minute)	0.339	0.199
Proteinuria (g/24 hours)	0.182	0.484
Renal SLICC score	-0.031	0.796
Total SLICC	-0.385	0.0053

^a^Significant results (*p *< 0.005) after the Bonferroni correction. C3, complement factor 3; C4, complement factor 4; CRP, C-reactive protein; EliA, fluorescence enzyme immunoassay test for anti-double-stranded DNA (Phadia GmbH); ELISA, enzyme-linked immunosorbent assay; ESR, erythrocyte sedimentation rate; SLE, systemic lupus erythematosus; SLEDAI, Systemic Lupus Erythematosus Disease activity index; SLICC, Systemic Lupus International Collaborating Clinics/American College of Rheumatology Damage Index for systemic lupus erythematosus.

**Table 4:** Multivariate models building on all significant factors in univariate analysis

Model	Feature	Unstandardised coefficients (B)	Standard error	Standardised coefficients beta	*t*	*p *value
1	(Constant)	74.907	23.552		3.180	0.003
	Anti-dsDNA (ELISA) Ab (U/ml)	0.413	0.149	0.381	2.765	0.008
2	(Constant)	72.028	23.730		3.035	0.004
	Anti-dsDNA (ELISA) Ab (U/ml)	0.520	0.184	0.480	2.828	0.007
	Anti-dsDNA Ab – EliA (IU/ml)	-0.142	0.142	-0.169	-0.997	0.324
3	(Constant)	68.464	26.533		2.580	0.013
	Anti-dsDNA (ELISA) Ab (U/ml)	0.512	0.187	0.472	2.732	0.009
	Anti-dsDNA Ab – EliA (IU/ml)	-0.137	0.145	-0.163	-0.947	0.349
	Total SLEDAI score	1.153	3.676	0.044	0.314	0.755
4	(Constant)	63.596	31.159		2.041	0.048
	Anti-dsDNA (ELISA) Ab (U/ml)	0.506	0.190	0.467	2.658	0.011
	Anti-dsDNA Ab – EliA (IU/ml)	-0.132	0.147	-0.158	-0.901	0.373
	Total SLEDAI score	1.013	3.743	0.039	0.271	0.788
	ESR (mm)	0.208	0.677	0.044	0.307	0.761
5	(Constant)	65.107	31.917		2.040	0.048
	Anti-dsDNA (ELISA) Ab (U/ml)	0.512	0.194	0.473	2.646	0.012
	Anti-dsDNA Ab – EliA (IU/ml)	-0.133	0.149	-0.159	-0.897	0.375
	Total SLEDAI score	1.137	3.808	0.044	0.299	0.767
	ESR (mm)	2.558 × 10^-03^	0.922	0.005	0.028	0.978
	CRP (mg/l)	0.154	0.522	0.057	0.295	0.769
6	(Constant)	61.031	58.443		1.044	0.303
	Anti-dsDNA (ELISA) Ab (U/ml)	0.513	0.196	0.473	2.614	0.013
	Anti-dsDNA Ab – EliA (IU/ml)	-0.128	0.164	-0.152	-0.780	0.440
	Total SLEDAI score	1.196	3.919	0.046	0.305	0.762
	ESR (mm)	8.484 × 10^-03^	0.956	0.002	0.009	0.993
	CRP (mg/l)	0.144	0.541	0.053	0.267	0.791
	Age	0.108	1.284	0.015	0.084	0.934
7	(Constant)	59.716	59.051		1.011	0.318
	Anti-dsDNA (ELISA) Ab (U/ml)	0.542	0.206	0.501	2.627	0.012
	Anti-DNA-EliA (IU/ml)	-0.134	0.166	-0.160	-0.808	0.424
	Total SLEDAI score	1.772	4.116	0.068	0.430	0.669
	ESR (mm)	-6.240 × 10^-03^	0.965	-0.001	-0.006	0.995
	CRP (mg/l)	0.144	0.546	0.053	0.264	0.794
	Age	-0.193	1.425	-0.028	-0.135	0.893
	Total SLICC	5.964	11.773	0.095	0.507	0.615

Ab, antibody; anti-dsDNA, anti-double-stranded DNA; CRP, C-reactive protein; EliA, fluorescence enzyme immunoassay test for anti-double-stranded DNA (Phadia GmbH); ELISA, enzyme-linked immunosorbent assay; ESR, erythrocyte sedimentation rate; SLEDAI, Systemic Lupus Erythematosus Disease activity index; SLICC, Systemic Lupus International Collaborating Clinics/American College of Rheumatology Damage Index for systemic lupus erythematosus.
